# Application of WHO International Biological Reference Standards to evaluate commercial serological tests for chronic Chagas disease

**DOI:** 10.1590/0074-02760200214

**Published:** 2020-07-24

**Authors:** Amadeo Sáez-Alquezar, Angela Cristina Verissimo Junqueira, Andressa da Matta Durans, André Valpassos Guimarães, José Abol Corrêa, D William Provance, Pedro Hernan Cabello, José Rodrigues Coura, Pedro Albajar Viñas

**Affiliations:** 1 Sociedade Brasileira de Análises Clínicas, Programa Nacional de Controle de Qualidade, Rio de Janeiro, RJ, Brasil; 2 Fundação Oswaldo Cruz-Fiocruz, Instituto Oswaldo Cruz, Laboratório de Doenças Parasitárias, Rio de Janeiro, RJ, Brasil; 3 Fundação Oswaldo Cruz-Fiocruz, Centro de Desenvolvimento Tecnológico em Saúde, Rio de Janeiro, RJ, Brasil; 4 Fundação Oswaldo Cruz-Fiocruz, Instituto Oswaldo Cruz, Laboratório Interdisciplinar de Pesquisas Médicas, Rio de Janeiro, RJ, Brasil; 5 Fundação Oswaldo Cruz-Fiocruz, Instituto Oswaldo Cruz, Laboratório de Genética Humana, Rio de Janeiro, RJ, Brasil; 6 Universidade do Grande Rio, Laboratório de Genética, Rio de Janeiro, RJ, Brasil; 7 World Health Organization, Department of Control of Neglected Tropical Diseases, Geneva, Switzerland

**Keywords:** Trypanosoma cruzi, human Chagas disease, serological diagnostic tests, immunoassays, International Biological Reference Standards

## Abstract

**BACKGROUND:**

Chagas disease, resulting from *Trypanosoma cruzi* infections, continues to be a health concern mainly in Latin American countries where the parasite is endemic. The laboratory diagnosis of a chronic infection is determined through serological assays for antibodies against *T. cruzi* and several tests are available that differ in key components, formats and methodologies. To date, no single test meets the criteria of a gold standard. The situation is further complicated by the difficulties associated with performance comparisons between different immunoassays or methodologies executed at different times and geographical areas.

**OBJECTIVE:**

To improve the diagnosis of Chagas disease, the WHO coordinated the development of two International Biological Reference Standards for antibodies against anti-*T. cruzi*: NIBSC 09/186 and NIBSC 09/188 that respectively represent geographical regions with the highest prevalence of TcII and TcI lineages of the parasite.

**METHODS:**

The principle goal of this study was to verify the behavior of these standards when assayed by several commercially available serological tests that employ different methods to capture and detect human anti-*T. cruzi* antibodies.

**FINDINGS AND MAIN CONCLUSIONS:**

The results reinforce the recommendation that these standards be considered for performance evaluations of commercialised immunoassays and should be an integral step in the development of new test components or assay paradigms.

Human Chagas disease is caused by an infection of the protozoa *Trypanosoma cruzi*, the etiological agent. First described by Carlos Chagas in 1909,^1^ to this day it still constitutes one of the main health problems in continental Latin America, where it is endemic and whose principal transmission is vector-borne by members of the triatomine family of insects.^2^ ^,^ ^3^ In the last decades, increased population movements have been observed between endemics to non-endemic areas, primarily immigration, resulting in a disease urbanisation phenomenon and an increase in the number of cases detected in the Northern hemisphere of the Americas, as well as in other continents.^4^ In the absence of an insect vector, the risk of transmission comes primarily from blood transfusion, organ transplants, congenital transmission and, with less frequency, laboratory accidents.^5^ ^,^ ^6^ According to the World Health Organization (WHO) and Pan American Health Organization (PAHO), in 2015 the worldwide number of infected persons range from 6 to 7 million persons with the majority living in Latin America, where more than 25 million are at risk of acquiring the disease.^2^ ^,^ ^7^ The incidence of Chagas disease beyond its historical geographical distribution has transformed it into a global public healthcare problem.^4^

It is of fundamental importance to accurately diagnosis *T. cruzi* infections through laboratory tests for the administration of the best course of patient treatment to curb disease progression and the prevention of disease transmission. Parasitological tests *for T. cruzi* infections can directly observe parasites in blood smears^3^ ^,^ ^8^ ^,^ ^9^ ^,^ ^10^ or after concentration techniques such as centrifugation, Strout method and microhematocrit. The detection of portions of the *T. cruzi* genome circulating in blood is also possible through molecular biology techniques.^3^ ^,^ ^11^ Blood smears are primarily reserved for diagnosing the acute phase or reactivation of an infection due to immunodepression, which corresponds to a high parasitaemia in the blood of infected individuals.^3^ ^,^ ^8^ ^,^ ^9^ ^,^ ^10^ In the transition to the chronic phase, the number of circulating parasites usually fall below the level of feasible detection through parasitological tests. While polymerase chain reaction (PCR) amplification can show greater sensitivity than parasitological tests, it still requires the capture of parasite nucleic acid in the patient sample for an accurate diagnosis.^9^ ^,^ ^11^ ^,^ ^12^ ^,^ ^13^ ^,^ ^14^ Its use as a diagnostic tool is further limited by the high costs of reagents and specialised equipment that require trained personnel and infrastructure as well as the absence of standardisation on a global scale.^15^ ^,^ ^16^

For the chronic phase, when parasitaemia is at its lowest level, serological assays offer an alternative diagnostic method by the detection of anti-*T. cruzi* antibodies. A variety of diagnostic tests have been developed and described in the literature and several of them have been commercialised.^17^ ^,^ ^18^ However, to date, no single test can be considered as a gold standard for diagnosis results and the recommendations in the Clinical Protocol and Chagas Therapeutic Guidelines is to employ a minimum of two different assays to confirm a diagnosis.^19^ ^,^ ^20^ The serological test format most frequently utilised for the screening of blood/blood products as well as organ transplantation donors and receivers to diagnosis infection is the enzyme linked immunosorbent assay (ELISA).^19^ Recently, chemiluminescent magnetic immunoassays (CMIA) has increasingly become an alternative to the ELISA format, among others, due to: compatibility with automation, less dependence on highly-trained and experienced personnel, scalability, digital read-out and a higher comparability rate among results. Each of these formats present different characteristics in relation to the antigenic targets employed, cutoff values and the type of apparatus used to perform measurements. In addition to these differences, direct comparisons of test performance are made more difficult by the dependence of calculations on sensitivity and specificity to the panel of patient sera used, which are often distinct and different between test evaluations.^19^

In 2007, the WHO organised a research group to develop biological resources representing the sera of individuals infected by *T. cruzi* at a scale that could be used as a reference to evaluate the performance of existing serological tests as well as the development of new tests. As a result, two regionally distinct samples were generated, defibrinated, aliquoted and lyophilised. After an extensive evaluation for their anti-*T. cruzi* antibody content, in 2011, these samples were established as WHO International Reference Standards or Biological References for the serological diagnosis of Chagas disease.^21^ One standard, NIBSC 09/186, is representative of a region with a prevalence for infections by the evolutionary lineage TcII that at the time of the collections was known to have five subtypes (TcII a-e)^22^ The other, NIBSC 09/188, was produced from sera collected within a geographical area endemic for lineage TcI. The purpose of the present work was to verify the behavior of these biological references using a diverse set of serological kits that employ different reagents and methodologies to detect anti-*T cruzi* antibodies within a single laboratory setting; a relevant assessment at the moment when, for the first time, Chagas disease immunoassays have been included in the WHO Model List of Essential In Vitro Diagnostics.^23^

## MATERIALS AND METHODS

*Biological references standard* - The WHO 1st International Reference Standards for Chagas disease antibody in Human Plasma^21^ were obtained from the WHO collaborating center at the National Institute for Biological Standards and Controls (Hertfordshire, UK). They consist of two freeze dried preparations, coded NIBSC 09/188 and NIBSC 09/186. NIBSC 09/188 contains anti-*T. cruzi* I (TcI) antibodies regionally collected from individuals living in Mexico. NIBSC code 09/186 contains anti-*T. cruzi* II (TcII, see discussion) antibodies regionally collected from individuals living in Brazil and Chile. Each standard was prepared and diluted as recommended using 0.5 mL distilled water to provide a 1:1 stock solution. A two-fold serial dilution series was generated from 1:2 to 1:64.

*Serological diagnostic kits* - Eight serological tests were included in the evaluation and each was conducted before their expiration dates (Table I). Six kits were an ELISA format (Gold; Bioschile; Biokit; D-Med; BioMérieux; Wiener 4.0), one used chemiluminescence with magnetic beads (CMIA; Abbot Architect) and one used a western blot format (TESA Blot; BioMérieux). The two BioMérieux kits are no longer commercially available.

Three ELISA kits utilised antigens of total lysates derived from cultured parasites. Three others utilised antigens consisting of recombinant proteins and one used a combination of lysate with recombinant proteins. The TESA blot is considered to be a complementary test consisting of a size fractionation of excreted and secreted antigen from trypomastigote cultures^24^ ^,^ ^25^ ^,^ ^26^ whose results are interpreted from a visual evaluation.

**TABLE I:** Details of the commercial serological tests employed for the application of the WHO International Biological Reference Standards

Commercial test	Method	Antigenic target	Country of origin	Batch	Reader/Analyser
Gold	ELISA	Lysate + Rec	Brazil	CHA084A	TECAN
Bioschile	ELISA	Lysate	Chile	1H110388	TECAN
Biokit	ELISA	Rec	Spain	L-1411	TECAN
D-med	ELISA	Lys	Argentina	110102	TECAN
Biomérieux	ELISA	Lys	France	1203106006	TECAN
Wiener	ELISA	Rec	Argentina	1109075160	TECAN
Abbott (Architect)	CMIA	Rec	USA	14857LI00	Architect i2000
Biomérieux (TESA blot)	WB	Ag Trypo	France	1204106150	N/A

ELISA: enzyme linked immunosorbent assay; CMIA: chemiluminescence magnet immunoassay; WB: Western blot; Lys: total *Trypanosoma cruzi* lysate; Rec: recombinant *T. cruzi* proteins; Ag Trypo: antigens excreted or secreted by trypomastigote forms of *T. cruzi*.

*ELISA assays* - Each commercial assay was used following the technical instructions provided by the manufacturer. ELISA plates were rinsed between steps using a Columbus Microplate Washer automated plate washer (TECAN, Männedorf, CH). For measurement of optical densities, a Sunrise™ ELISA reader was used (TECAN). For the chemiluminescent magnetic immunoassay, an Architect i2000 (Abbott, Illinois, USA) was employed.

*Statistical analysis* - The statistical analyses consisted of a Two-Way ANOVA Without Replication that was defined using as the variation factors the degree of dilution and the manufacturer of the test.^27^ ^,^ ^28^ The coefficient means [optical density (OD)/cutoff (CO)] were organised in the form of a two-dimensional matrix that was analysed through a Two-Way variance analysis (Dilution x Manufacturer) to measure the relative effects of these two factors.^27^ ^,^ ^28^ The inference of significance of the results was assessed at a p value ≤ 0.05. The compilation, organisation and tabulation of the data were accomplished using the IBM SPSS 22.0 Software (https://www.ibm.com/analytics/spss-statistics-software) and Microsoft Excel 2013 (https://www.microsoft.com /en).

## RESULTS

The performance of the two WHO commissioned international biological reference standards for anti-*T. cruzi* antibodies, NIBSC 09/186 and NIBSC 09/188, was evaluated by the ability of a set of commercially available assays, which are approved by multiple regulatory agencies for the diagnosis of chronic Chagas disease, to detect and measure their respective pool of *T. cruzi* specific antibodies (Table I). The standards represent the immunological response in three endemic regions, Mexico, Brazil and Chile, to infections by two lineages of *T. cruzi*, TcI and TcII, and are organised by the lineages. NIBSC 09/188 contains antibodies that are predominantly generated against Tc1 from individuals living in Mexico. NIBSC 09/186 contains antibodies primarily against TcII from individuals living in Brazil and Chile.

The results for each test are presented in Tables II and III as the ratio of the OD to assay CO values, except for the CMIA assay where the equivalent to optical density was relative light units. Converting the data into a ratio normalised the differences in the absolute values and cutoffs between tests, wherein a value greater than 1.0 was considered reactive (positive) and below 1.0 was considered non-reactive (negative). A homogeneity test showed significant differences in the results related to higher dilutions of the International Biological References Standards and the commercial tests, which are shown as the p value in Tables II and III.

From a graphical representation of the data, shown in Figure, an apparent grouping of the assay kits based on their sensitivity could be discerned. For NIBSC 09/188 (Panel A), three different groups could be distinguished that began with the lowest dilution level and continued through to a dilution of 1:16. At the next highest dilution (1:32), only two kits were sufficiently sensitive to display reactivity. No assay kit displayed activity at the highest dilution of the biological reference standard (1/64). The reactivity of all kits was lower against NIBSC 09/186 (Panel B), which could be visually segregated into two groups. Only one assay showed reactivity at a dilution of 1/32 (CMIA). The detection limit of each commercial test is summarised in Table IV.

**TABLE II:** Immunoassay results for a serial dilution of WHO Biological Reference NIBSC 09/188 (TCI) normalised as the ratio of optical density (OD) to cutoff (CO) value

NIBSC 09/188 Dilution	Methodology							
	ELISA						CMIA^*^	Homogeneity test
	Gold	BiosChile	Biokit	D-Med	BioMérieux	Wiener	Abbott	p
1/1	7.1	3.3	3.4	6.3	3.3	9.9	10.8	0.132
1/2	5.9	2.8	1.3	5.0	2.2	8.2	9.5	0.096
1/4	4.3	2.2	0.6	3.8	1.7	6.4	7.7	0.054^**^
1/8	2.7	1.6	0.3	2.7	1.3	3.8	6.2	0.009^***^
1/16	1.6	1.3	0.2	1.8	0.7	2.1	3.6	0.000^***^
1/32	1.4	1.0	0.2	1.0	0.2	0.9	2.1	0.000^***^
1/64	0.8	0.8	0.2	0.5	0.1	0.3	0.8	0.000^***^

ELISA: enzyme linked sorbent assay; CMIA: chemiluminescence magnet immunoassay. *: measurements are relative light units, not OD; **: significant difference; ***: border line to significance.

**TABLE III:** Immunoassay results for a serial dilution of WHO Biological Reference NIBSC 09/186 (TCII) normalised as the ratio of optical density (OD) to cutoff (CO) value

NIBSC 09/186 Dilution	Methodology							
	ELISA						CMIA^*^	Homogeneity test
	Gold	BiosChile	Biokit	D-Med	BioMérieux	Wiener	Abbott	p
1/1	8.0	2.8	3.6	7.3	3.9	8.3	8.8	0.379
1/2	6.2	2.2	1.0	4.7	2.8	6.4	7.4	0.163
1/4	3.8	1.8	0.5	2.9	2.0	4.3	6.9	0.029^**^
1/8	2.4	1.2	0.2	1.6	1.0	2.3	4.7	0.009^**^
1/16	1.2	0.8	0.2	0.9	0.5	1.4	2.4	0.000^**^
1/32	0.5	0.4	0.2	0.4	0.1	0.7	1.1	0.000^**^
1/64	0.4	0.4	0.2	0.4	0.0	0.3	0.5	0.000^**^

ELISA: enzyme linked sorbent assay; CMIA: chemiluminescence magnet immunoassay. *: measurements are relative light units, not OD; **: significant difference.

**Figure:**
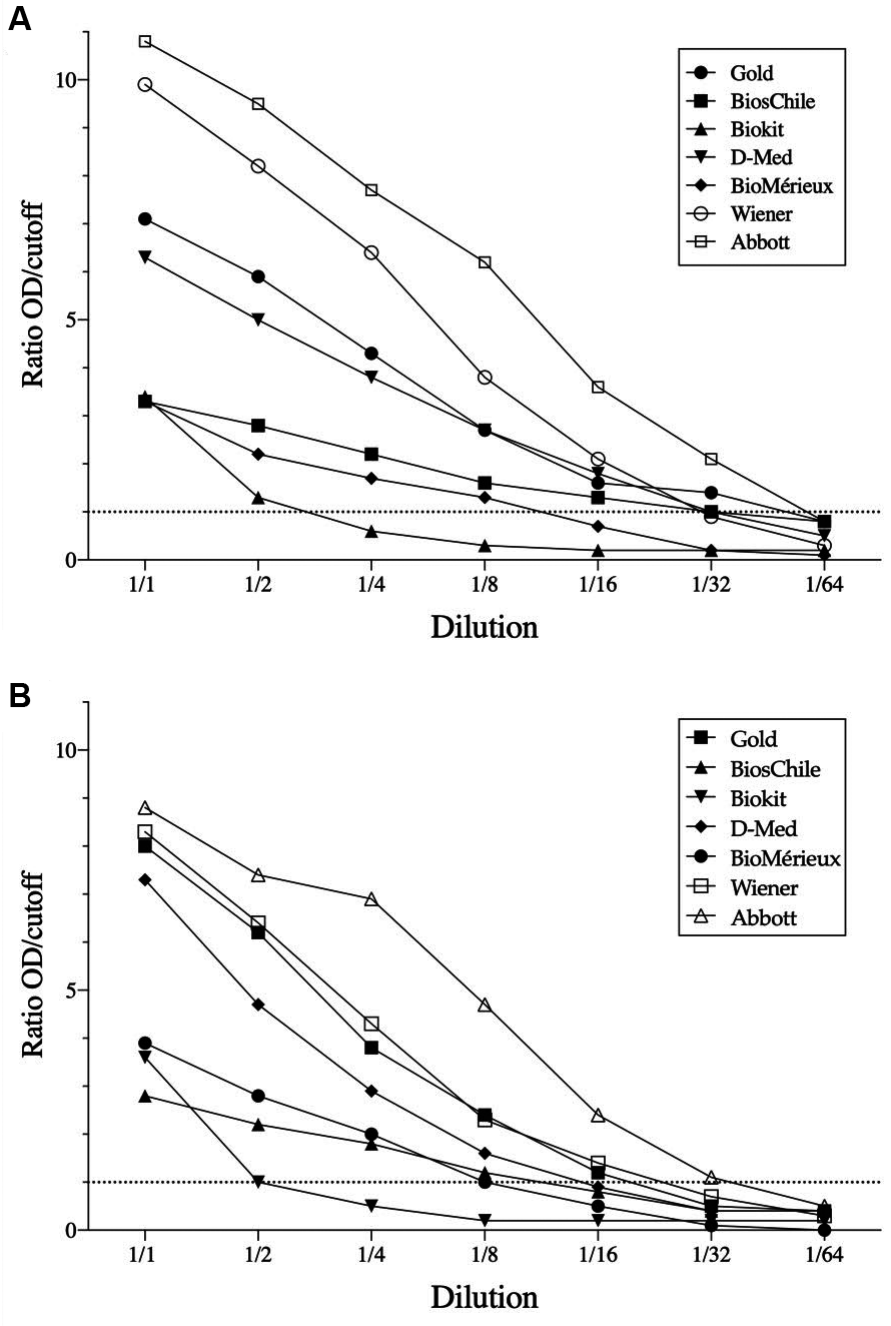
Application of the WHO International Biological References Standards for Chagas disease to commercial diagnostic assays. A dilution series of NIBSC 09/188 (Panel A) and NIBSC 09/186 (Panel B) was applied to the commercial kits listed in Table I. Data represent the mean from three independent experiments.

**TABLE IV:** Detection limits of the immunoassays for the detection of anti-*Trypanosoma cruzi* antibodies in the WHO International Biological Reference Standards

Dilution	Commercial tests	
	WHO/NIBSC 09/186	WHO/NIBSC 09/188
1/2	BioKit	BioKit
1/8	BioChile - D.MED - BioMerieux	BioMerieux
1/16	Gold - Wiener	Wiener
1/32	Abbott	Gold - BioChile - D.MED - Abbott

The significance of the results between test formats was further analysed by a Two-Way ANOVA Without Replication test. There were significant differences between the commercial kit results and also in relation to dilutions in both NIBSC 09/186 (Table V) and NIBSC 09/188 (Table VI). In this type of analysis, the source of internal variation will be equal to zero. Therefore, the total variation must correspond to the variation among all observations, which can be broken down into three parts: 1. variation that depends on the effect related to the different serological tests; 2. variation due to the effect of each dilution level of the biological reference standards; 3. the residue, that is, variation independent of the kits and the dilutions. This last component of the variation is the basis for testing the effects related to the two factors of test manufacturer and sample dilution. As expected, the different dilutions of the reference standards had the most significant impact on the test results as represented by the p-values that were effectively zero. The individual tests also showed a statistically significant difference in performance for each of the biological reference standards.

In the absence of a gold standard diagnostic test, a second test is recommended to confirm the first test results. Due to its higher specificity, a TESA blot was also used to evaluate the two biological reference standards. This is a visually scored test for the detection (+) of specific protein bands in the range of 120 kDa to 200 kDa. The TESA blot showed the expected bands, although at a lower dilution than the commercial kits that is consistent with the lower sensitivity of this assay (Table VII).

**TABLE V:** Two-way analysis of variance (ANOVA) analysis for the application of the WHO International Biological Reference Standard (WHO/NIBSC 09/188)

Two-way ANOVA of optical density/cutoff averages (test and dilution)					
WHO/NIBSC 09/188					
Source of variation	SS	DF	MS	F-value	*p*-value
Assay kit	131.92	6	21.99	9.18	0.002
Dilution	195.27	6	32.54	13.59	0.000
Residual	86.18	36	2.39		
Total	413.37	48	8.61		

DF: degree of freedom; MS: medium square; SS: sum of squares.

**TABLE VI:** Two-way analysis of variance (ANOVA) analysis for the application of the International Biological Reference Standard (WHO/NIBSC 09/186)

Two-way ANOVA of optical density/cutoff averages (test and dilution)					
WHO/NIBSC 09/186					
Source of variation	SS	DF	MS	F-value	*p*-value
Assay kit	73.55	6	12.26	6.22	0.013
Dilution	196.86	6	32.81	16.65	0.000
Residual	70.94	36	1.97		
Total	341.34	48	7.11		

DF: degree of freedom; MS: medium square; SQ: sum of squares.

**TABLE VII:** Performance of the TESA blot by BioMérieux to detect anti-*Trypanosoma cruzi* antibodies in WHO International Biological References over a dilution series

TESA blot		
Dilution	NIBSC 09/188	NIBSC 09/186
1/1	(+)	(+)
1/2	(+)	(+)
1/4	(+)	(+)
1/8	(+)	(+)
1/16	(-)	(-)
1/32	(-)	(-)
1/64	(-)	(-)

## DISCUSSION

In the last decade, for the first time the WHO coordinated the development of biological references for use in immunoassays to detect antibodies against *T. cruzi* in the diagnosis of chronic Chagas disease. The results of the comparative performance of various assays currently commercialised using the biological references supports the recommendation for their consideration in the evaluation of tests as well as for the testing of prototypes under development.

In serological tests, the objective is for the final result, represented here as the ratio of the measurement of the signal intensity to cutoff, to strictly relate to the diagnostic status of the patient. However, the diverse elements that comprise an assay also contribute to the final result. These factors include the design of the assay, the target antigens, the platform, instrumentation and accessory components such as anti-human secondary antibodies, their conjugated enzymes, enzyme substrates and buffers. With all of these contributing variables, the final value does not exclusively correspond to the actual concentration of the antibodies under analysis in the sample. This limits serological tests to being qualitative assays where the measured intensity can only indicate the presence or absence of reactive antibodies. The qualitative nature of serological testes makes it is very difficult to compare the results obtained with kits of different origins and/or methodologies.

In the study originally describing the International Biological References Standards,^21^ their performance was evaluated against several commercial and “in-house” tests of different methodologies by 24 laboratories located in 16 countries, which confirmed their ability to distinguish between tests with different sensitivities. Each ampule of biological reference material contains the equivalent of 0.5 mL of lyophilised plasma that was defined to correspond to 0.5 IU of reactivity. The employment of a serial dilution permits the association of a specific numeric unit to the test result related to the highest dilution factor that displayed reactivity and is represented its reciprocal. This provides relatable information on the relative strength of the immunoassay evaluated.

To remove any contribution of the laboratory setting to the final result, a set of commercial tests were executed in a single laboratory using the biological reference standards. For the ELISA tests executed here, the relative strengths for NIBSC 09/186 were observed to be between 8 and 16 with only one showing a relative strength of 32, which was obtained with the CMIA format. In comparison, a higher reactivity was measured for each assay over the dilution series of NIBSC 09/188 with four test kits showing a relative strength of 32, including the CMIA format. Overall, each of the tests showed a fairly linear relationship between the dilution value and reactivity, which suggested good consistency between each measurement.

The difference in the relative strengths of each test to the two different standards highlights a major difficulty associated with the development of a gold standard test for detecting *T. cruzi* antibodies; the diversity in the geographical distribution of parasite lineages and the corresponding immunological response. Only one immunoassay displayed reactivity for NIBSC 09/186 at a dilution of 1/32 whereas four immunoassays showed reactivity at this dilution for NIBSC 09/188. No assays showed reactivity at the highest suggested dilution of 1/64. As the reactivity of each test is statistically significant to the result and their relative strength differed between the standards, it strongly suggests that NIBSC 09/186 and 09/188 have a different composition of anti-*T. cruzi* antibodies.

At the time that the sera used to generate the biological references was collected, there were only two recognised lineages, TcI and TcII, which were used to define the geographical regions used to differentiate the two biological references NIBSC 09/188 and 09/186, respectively. Due to the high degree of intraspecific *T. cruzi* polymorphism, seven distinct lineages, called Discrete Typing Units (DTU), TcI to TcVI and Tcbat have since been defined.^29^ ^,^ ^30^ ^,^ ^31^ ^,^ ^32^ ^,^ ^33^ ^,^ ^34^ ^,^ ^35^ With the nomenclature change and the increase in lineages, the five subgroups of TcII (TcII a-e)^22^ were designated as the independent DTUs TcII-TcVI.^29^ ^,^ ^30^ ^,^ ^31^ ^,^ ^32^ This would suggest that NIBSC 09/186 most likely represents a greater diversity of lineages that could effectively dilute the specific antibodies against each lineage and account for the reduced sensitivity observed for most of the tests analysed. However, the biological references together were intended to contain antibodies generated against all lineages of *T. cruzi*, irrespective of their distribution between the two and the relative concentration of antibodies to common epitopes should be nearly equivalent. Unless there is a diminished immunological reaction against infections by TcII-TcVI than TcI that would reduce the antibody titer in NIBSC 09/186 compared to NIBSC 09/188, the difference in the results would suggest that the antigenic compositions in the different assays are more representative of TcI than TcII-TcVI.

This difference in antigen composition is supported by the results with the CMIA platform that appeared to be equally sensitive to the two regionally representative biological standards, although the result with NIBSC 09/186 at the 1/32 dilution was close to being defined as non-reactive. Its higher sensitivity was evident by the consistently higher values observed for the CMIA-based assay throughout the serial dilution compared to the other tests. The results suggest that the CMIA assay can serve as a blood screening platform with the lowest possible rate of false negative results, which is a public health objective that drove its development.

The TESA blot showed the lowest sensitivity for both biological references with reactivity observed only up to the 1/8 dilution. Considering that the TESA blot is primarily a complementary test for specificity to a previously reactive test, the lower sensitivity can be accounted for during execution. Unlike the ELISA formats, the western blot format of the TESA blot did not show a difference in sensitivity to NIBSC 09/186 & 09/188 suggesting that its application in the diagnosis of Chagas disease can be universal.

Regardless of the serological panels used or the populations studied, we believe that it is essential to have a mechanism to be able to compare the results obtained with different immunoassays and methodologies. The WHO International Biological References Standards can serve this mechanism to evaluate the performance of all commercialised immunoassays and prototypes under development to meet the ongoing need for a gold standard test to diagnose human Chagas disease.
